# Perception of Life-Control Is Associated With Medical Care Satisfaction in Chronically Ill Rural Older Adults

**DOI:** 10.1093/geroni/igaa057.1452

**Published:** 2020-12-16

**Authors:** Anne Halli-Tierney, Hyunjin Noh, Lewis Lee

**Affiliations:** The University of Alabama, Tuscaloosa, Alabama, United States

## Abstract

Prior studies show patient populations have varied experiences with healthcare systems, and this may influence satisfaction with medical care. Patients feeling control over life circumstances may have resilience and ability to adapt to adverse situations. Given socioeconomic and medical differences in older adults we examined factors influencing satisfaction with medical care in the setting of chronic conditions and pain. 100 older adults in rural West Alabama with chronic illnesses and pain were recruited from community senior centers and interviewed with a structured questionnaire. Participants were queried about medical interventions for chronic conditions and satisfaction with medical care. Overall assessment of life-control was measured by the West Haven Yale Multidimensional Pain Index (WHYMPI). Bivariate correlation and multivariate analysis were conducted. Correlations between satisfaction with medical intervention and life-control scores were significantly positive (r=.21, p<.05). Satisfaction with medical intervention and other covariates explained approximately 14.1% variance in life-control scores, R2=.141. Multivariate regression results confirmed those highly satisfied with medical intervention were marginally significantly likely to have increased life-control scores, b=.20, SE=.12, p=.088. Married persons were more likely to have higher life-control scores than those with other marital statuses, b=.84, SE=.34, p<.05. Income was positively associated with life-control scores, b=.18, SE=.08, p<.05. Older adults may perceive greater satisfaction from medical care if they feel greater control over life circumstances. Socioeconomic factors (marital status, income) are associated with life control. These findings can help predict satisfaction with healthcare and find ways to make healthcare more accessible to all.

